# Low colostrum intake results in potential accumulation of peroxisome lipid substrates in vaginal tissue of 3-week-old gilts

**DOI:** 10.1242/bio.060044

**Published:** 2023-08-11

**Authors:** Kayla Mills, Jocelyn Sheets, Kelsey Teeple, Adrianna Mann, Aridany Suarez-Trujillo, Kara Stewart, Christina Ferreira, Theresa Casey

**Affiliations:** ^1^ US Department of Agriculture, Agricultural Research Service, Beltsville Agricultural Research Center (BARC), Beltsville, MD 20705, USA; ^2^ Department of Animal Science, Purdue University, West Lafayette, IN 47907, USA; ^3^ Department of Animal Science, Berry College, Mount Berry, GA 30149, USA; ^4^ Metabolite Profiling Facility, Bindley Bioscience Center, Purdue University, West Lafayette, IN 47907, USA

**Keywords:** Colostrum, Lipidome, Development, Gilt

## Abstract

Low colostrum intake relates to poorer health and infertility in swine. We previously connected vaginal lipid profiles at weaning to fertility of sows. We hypothesized vaginal lipidome varied with colostrum intake. Our objective was to determine whether indicators of colostrum intake, immunocrit (IM) and weight gain 24 h postnatal (PN), related to vaginal lipids at d21 PN. Gilts (*n*=60) were weighed and blood sampled to measure IM. On d21 PN vaginal swabs were taken and lipids measured using multiple reaction monitoring. Abundance of multiple lipids differed (*P*<0.05) between gilts categorized as high versus low IM and high versus low 24 h gain. The abundance of multiple lipids correlated with IM and 24 h gain. Phosphatidylcholine PC(36:3), PC(36:2), and arachidonic acid (C20:4) positively (*P*<0.05) correlated with IM. The ether lipid PCo(38:6) and multiple cholesteryl esters negatively (*P*<0.05) correlated with IM. ROC analysis indicated arachidonic acid and docosanoic acid (C22:0) may serve as excellent biomarkers that distinguish between high and low IM. Similar to gilts found to be infertile, lipid profiles of low colostrum intake animals had greater abundance of very long chain fatty acids, lipids with high levels of unsaturation, and cholesteryl esters, which are metabolized in peroxisomes indicating their potential dysfunction.

## Teaser

**Summary:** Upon birth, neonates ingest colostrum and the level of intake is related to long-term health and development. We report that gilts with low levels of colostrum intake have greater abundance of lipids metabolized in peroxisomes, which may indicate inadequate initiation of beta-oxidation to catabolize fats.

## INTRODUCTION

Perinatal nutritional exposures establish trajectories of growth and development that are carried through to maturity, and reflect long-term phenotype including tissue composition, feed efficiency and reproductive performance of swine (Bartol et al., 2013; Oster et al., 2012; Casey et al., 2018b; Suarez-Trujillo et al., 2019). During lactation, the mother delivers time-dependent signals through milk that regulate and program growth and development of the neonate. Studies in swine demonstrate that ingestion of maternal milk affects nearly every organ of the body with morphological and gene expression differences in gastrointestinal tract, liver and the female reproductive tract between piglets fed formula versus sow milk (Bartol and Bagnell, 2012; Ronis et al., 2011).

The window of opportunity for milk-borne factors to influence neonate development, termed lactocrine signaling, is limited, and primarily occurs prior to closure of tight junctions between the cells lining the piglet's gut. Closure of the gut occurs by 48 h postnatal (Bartol et al., 2009). Colostrum is the first secretion from the mammary gland and available to neonatal swine within the first 24 h postnatal. Colostrum is distinct from mature milk, and is a highly concentrated secretion of nutrients, immune, and bioactive factors (Peaker, 2002; Dividich et al., 2005). Ingestion of colostrum sets the developmental trajectory of reproductive competence in swine, and like human infants, also affects body composition (e.g. lean body mass) at maturity (Bagnell and Bartol, 2019; Ferrari et al., 2014; Quesnel et al., 2012; Gancarcikova et al., 2008; Devillers et al., 2011). The level of colostrum intake is also a primary driver of boar fertility and directly related to the total number of sperm per ejaculate (Flowers, 2022). Similarly, the level of colostrum intake by gilts affects her age at attainment of puberty, number of ovulations and milk production during lactation (Vallet et al., 2015).

Identifying gilts that ingest adequate amounts of colostrum would help in the selection of replacement animals in the sow production herd. Ingestion of colostrum can be estimated by the 24 h immunocrit (IM) ratio, which measures precipitated immunoglobulins from neonate blood plasma. Colostrum ingestion can also be estimated by 24 h weight gain, and piglets that ingested at least 20% of their birthweight in colostrum over the first 24 h postnatal were on a higher plane of growth than piglets that ingested less (Devillers et al., 2011). However, the sensitivity and specificity of 24 h weight gain and immunocrit are relatively low. Analysis of data from Vallet and others (Vallet et al., 2015) indicates an estimated predictive value of the immunocrit ratio for age at first estrus of approximately 41%. We conducted several studies to explore if there were any associations between lipidome of biological material collected from vaginal swabs and perinatal nutrition (Harlow et al., 2019a,b; Casey et al., 2018a). Comparison of vaginal swab lipid profiles between gilts that suckled colostrum versus those fed milk replacer, indicated that a glycerolipid containing arachidonic acid (C20:4) was a potential biomarker for colostrum intake (Casey et al., 2018a). In a follow-up study, comparison of vaginal lipidomes between gilts fed milk replacer versus those that suckled colostrum found distinct vaginal lipidomes between gilts groups on PN d2 (Harlow et al., 2019a,b). We then investigated if there was a relationship between the vaginal lipidome on PN d21 and long-term fertility in a longitudinal study. A similar set of lipids distinguished between gilts that were formula fed versus colostrum fed on PN d2 and those sampled on PN d21 and distinguished between animals found to be infertile versus highly fertile production sows (Mills et al., 2021). The overall aim of the study described here was to determine if the level of colostrum intake of gilts raised within their litters was reflected in their vaginal lipidome at weaning. We measured immunocrit (IM) and 24 h gain of gilts and determined the relationship of these markers of colostrum intake to relative abundance of lipids isolated from swabs of vaginal tissue on PN d21.

## RESULTS

### Vaginal lipidome differences between gilts categorized by high and low IM and 24H weight gain

Gilts were selected from the litters of eleven sows. Birth litter size ranged from five to 18 piglets per sow, with a mean of 14 piglets per litter. There was a significant relationship (r=0.72, *P*<0.05) between litter size and average birth weight of gilts in the litter. However, this relationship was almost entirely driven by sow H8, which had five piglets, so when the sow-litter was removed the relationship was lower (r=0.4).

**Fig. 1.**
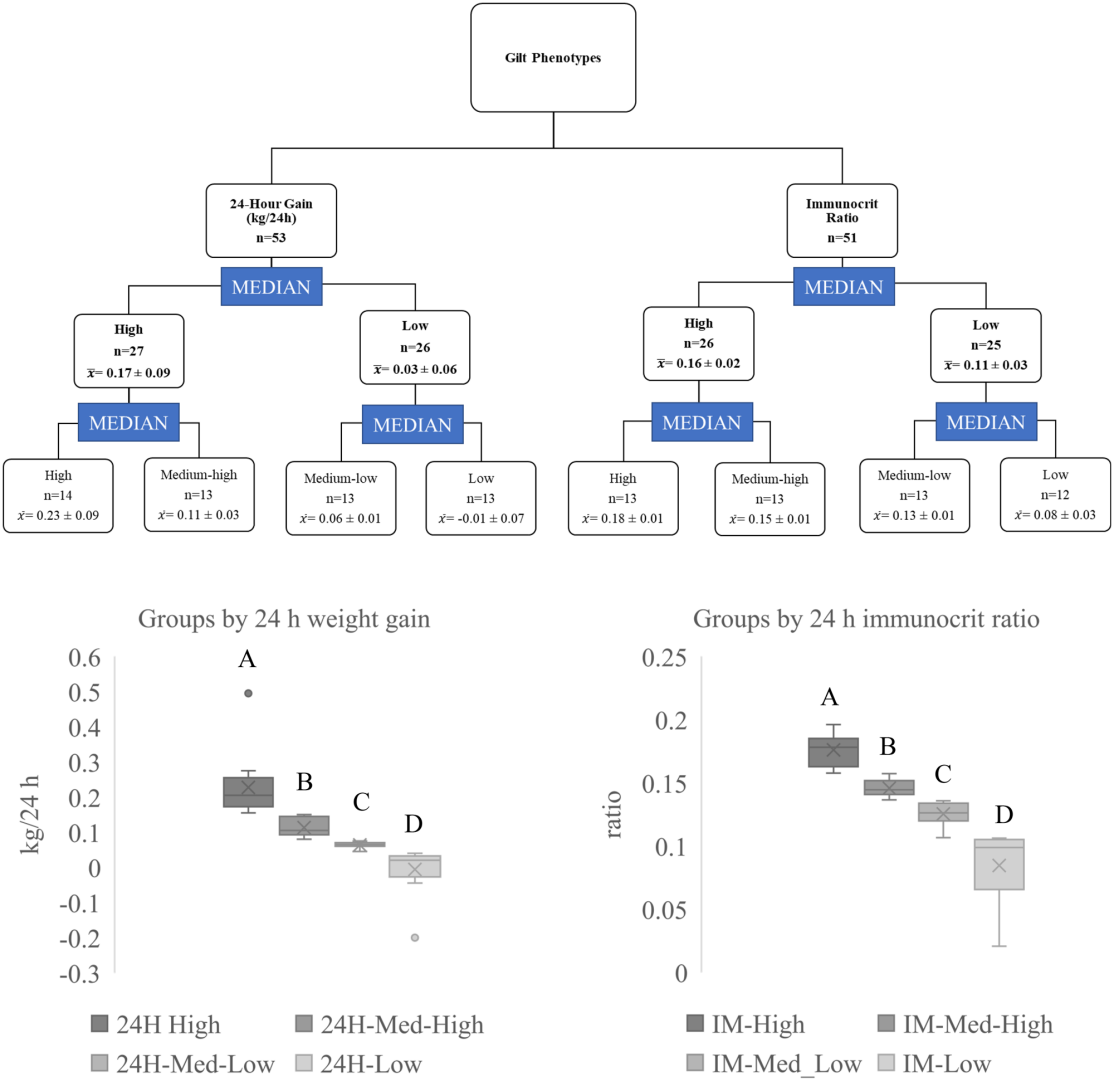
**Categorization of gilts by 24 h weight gain and immunocrit ratio.** (A) Schematic of assignment of gilts into high and low and intermediate categories of medium–high and medium–low of 24 h weight gain and immunocrit ratio. For the 24 h gain, there were negative values for the low gain group as they lost weight during this period. Box and whisker plots of gilts within each category of (B) 24 h weight gain (*n*=53) and (C) immunocrit ratio (IM; *n*=51). Differing letters indicate a difference at *P*<0.05.

Gilt birthweight was significantly related to IM (*P*<0.0001; r=0.64), but there was no relationship (r=0.13) between birth weight and 24 h gain. To distinguish between animals that varied in their perinatal nutritional environment, gilts were assigned to one of four groups based on IM or 24 h weight gain (Fig. 1). Although there was a significant (*P*=0.05) but weak (r=0.25) relationship between IM and 24 h gain, the categorization of gilts into low IM (*n*=12) and high IM (*n*=13) and low 24 h gain (*n*=13) and high 24 h gain (*n*=14) resulted in eight distinct groups and two distinct comparisons. Three animals overlapped between IM-high and 24 h-high gain, and five animals overlapped between IM-low and 24 h-low gain groups. There was also one animal categorized as high IM but low 24 h gain, and similarly one animal categorized as low IM but high 24 h gain.

The rapid screening of lipids by mass spectrometry was performed using the multiple reaction monitoring (MRM) profiling method on lipids previously identified as differentiating for colostrum versus formula feeding (method 1-M1 and 2-M2; Harlow et al., 2019a) and fertility outcome (Mills et al., 2021), including free fatty acids (FFA). Another distinct analysis of phosphatidylcholine and sphingomyelin classes (PCSM) of lipids was also run. The attributions are tentative and based on the Lipid Maps database (Reis et al., 2023). Lipids that distinguished between low and high IM and low and high 24 h weight gain are listed in Table 1. Within the M1 analysis, eight lipids were found different between high and low IM gilts and 35 were different between high and low 24 h weight gain. In the M2 analysis, four triacylglycerols (TAG) were found more abundant in the IM high versus low group. The number of total carbons across the three fatty acyl groups of these TAG MRM varied from 50-52, with one of the fatty acids being 16 or 18 carbons in length with no unsaturated bonds. Only one of the TAG had a fatty acid with a mono-unsaturation, and all others were saturated. Within the PCSM analysis, the abundance of 29 lipids differentiated between gilts with high and low 24 h gain, and 28 of these were more abundant in low 24 h gain group. In the FFA analysis, C20:4 (arachidonic acid) was found significantly more abundant in IM high, and tended to be greater in high 24 h gain group versus low. Pearson's correlation analysis was used to identify lipids that varied in abundance in relation to IM or 24 h weight gain across all animals. Multiple lipids showed positive or negative relationships with these variables and are listed in Tables 2 and 3, respectively.Table S1

**Table 1.**
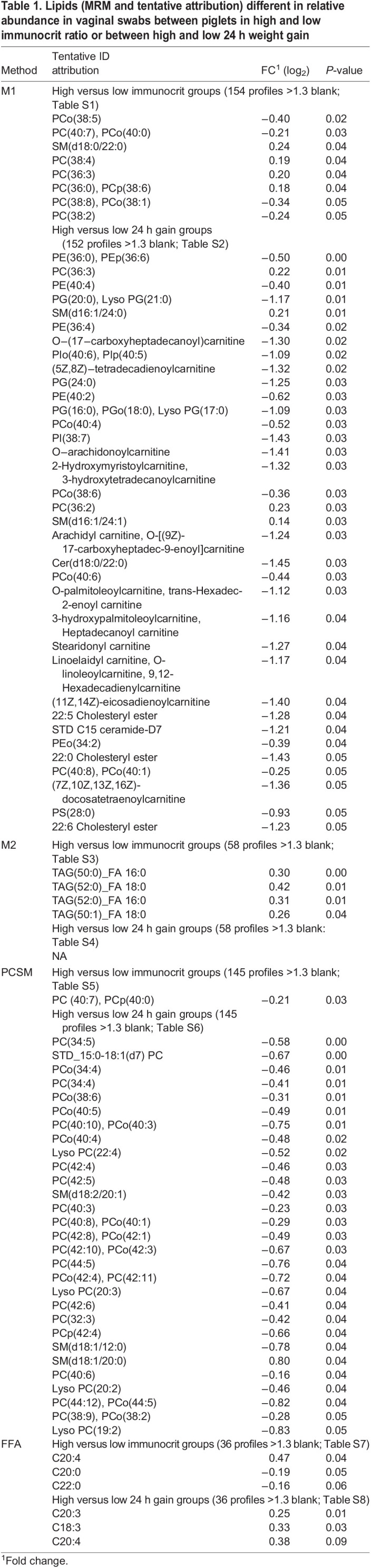
Lipids (MRM and tentative attribution) different in relative abundance in vaginal swabs between piglets in high and low immunocrit ratio or between high and low 24 h weight gain

**Table 2.**
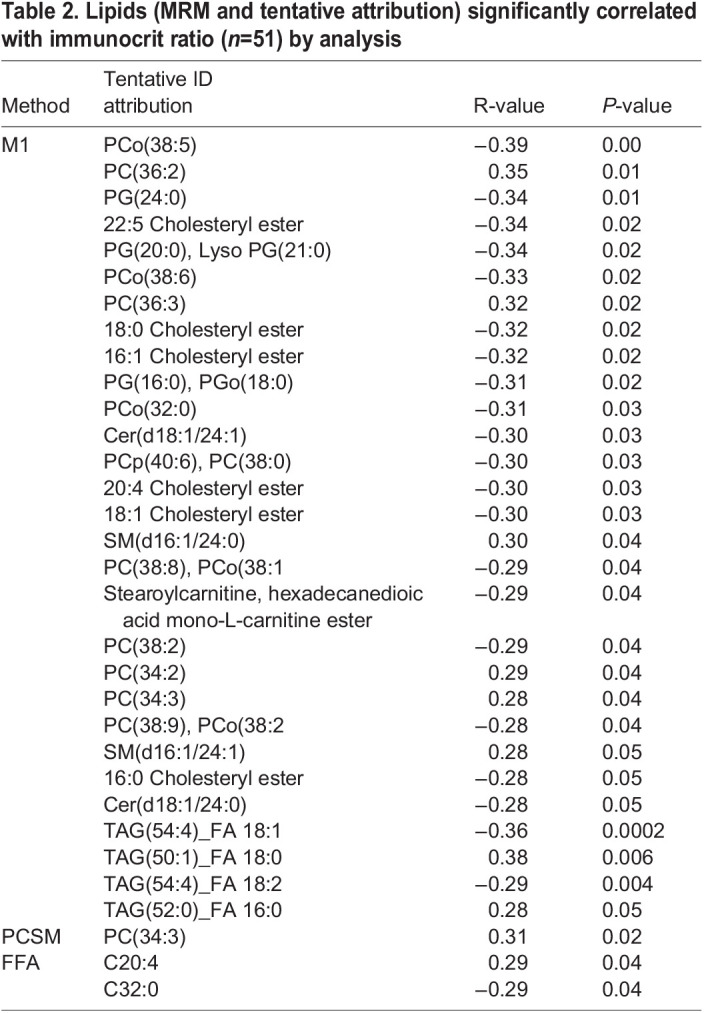
Lipids (MRM and tentative attribution) significantly correlated with immunocrit ratio (*n*=51) by analysis

**Table 3.**
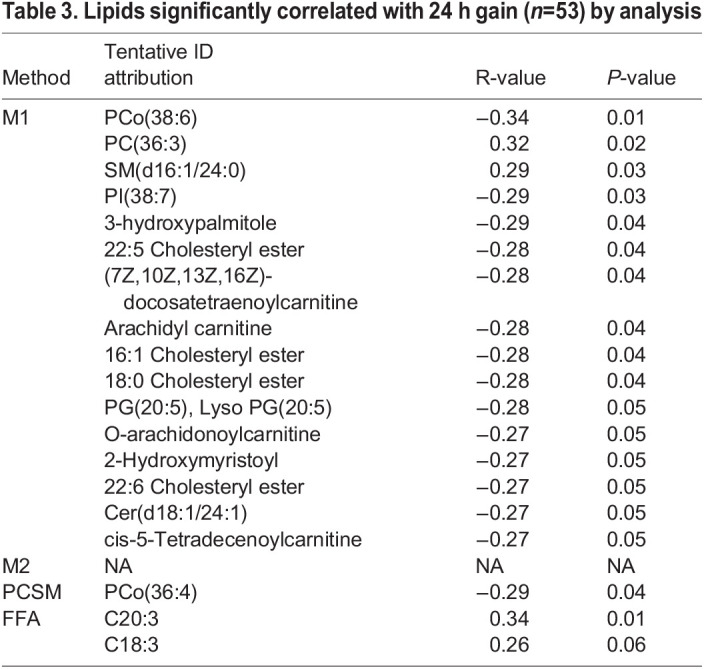
Lipids significantly correlated with 24 h gain (*n*=53) by analysis

**Fig. 2.**
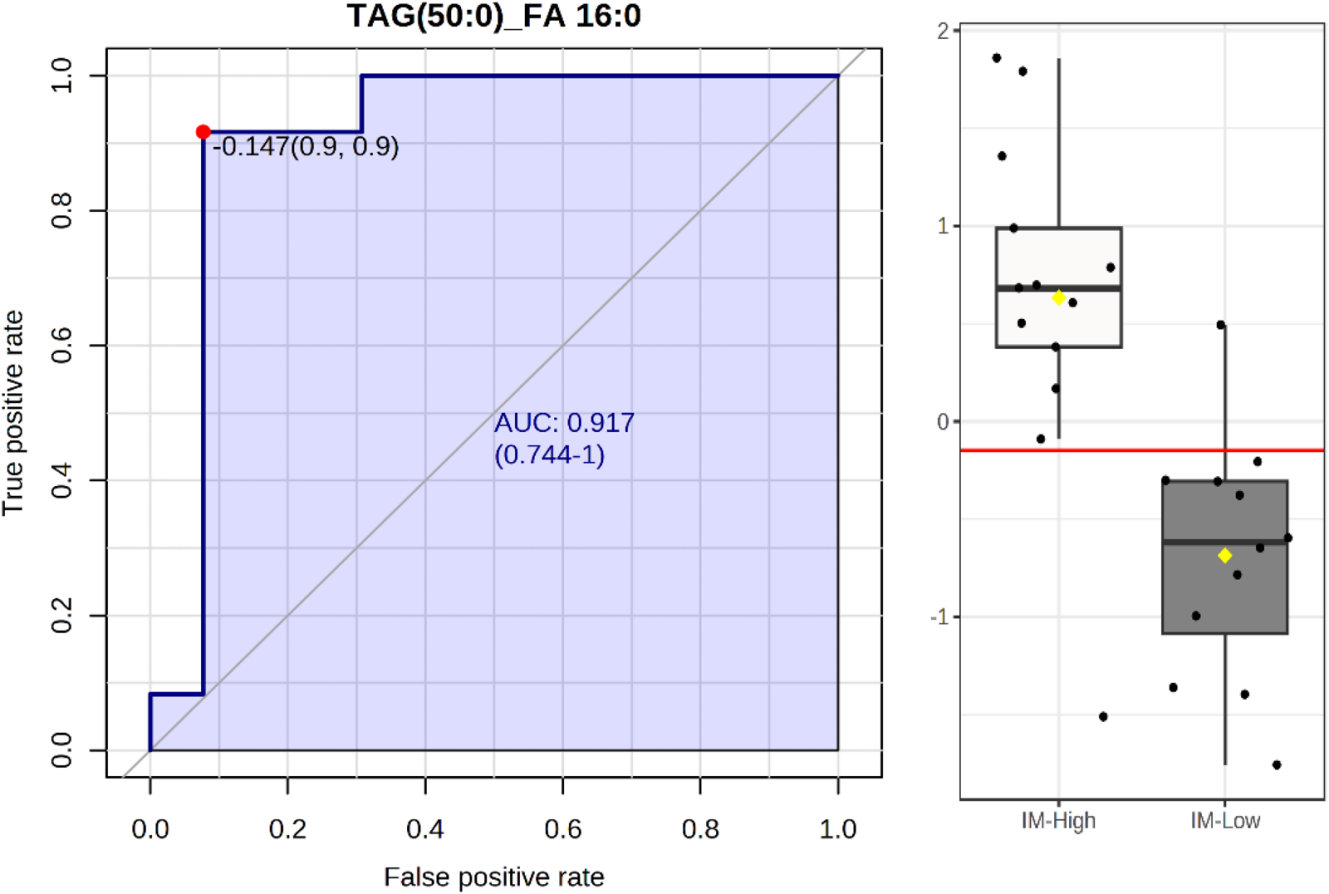
Univariate ROC analysis indicated TAG(50:0)_FA16:0 has the potential to be an excellent (AUC=0.917) biomarker to distinguish between IM high (*n*=13) and low (*n*=12) gilts.

**Fig. 3.**
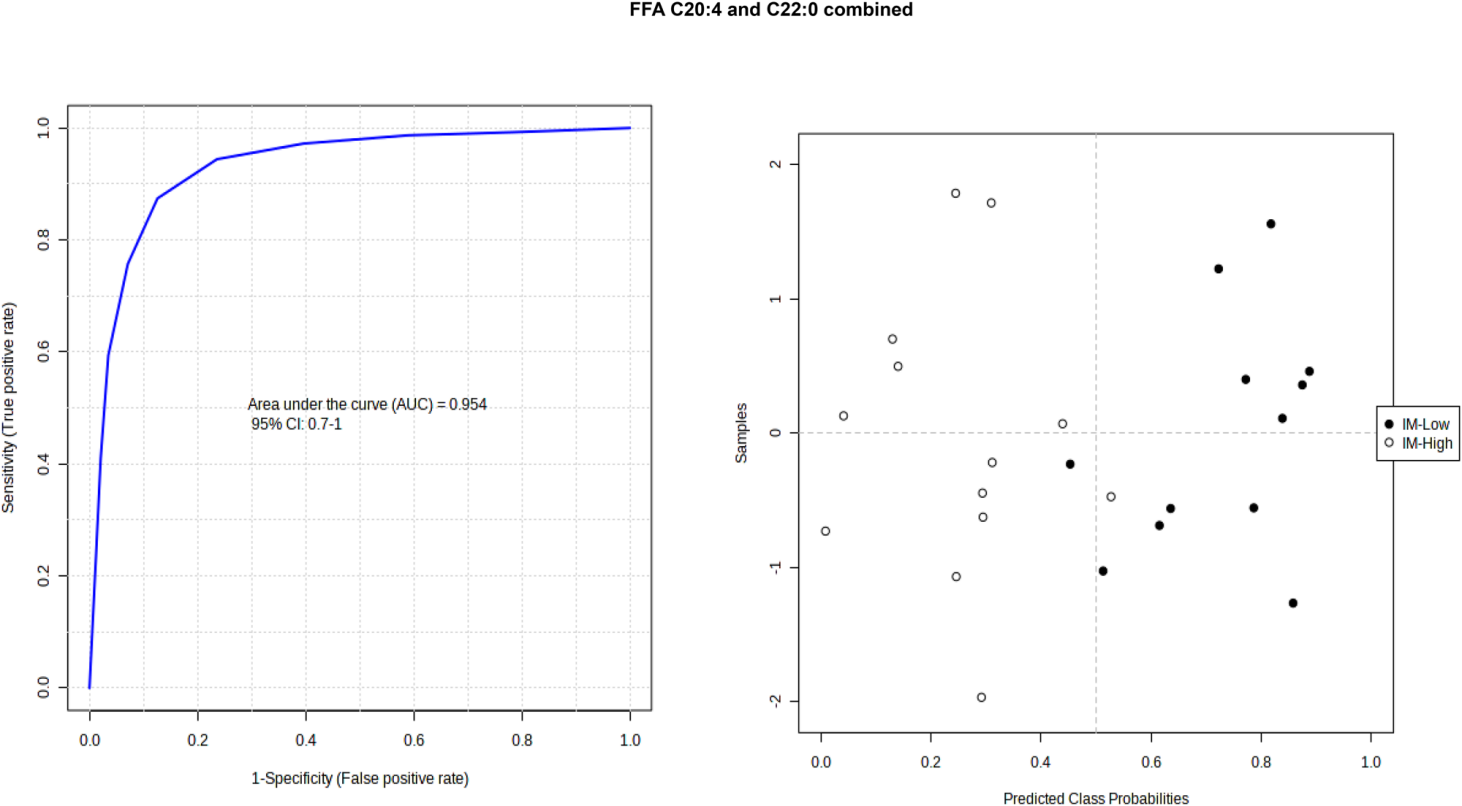
**Combining lipids for ROC analysis of potential biomarkers in the FFA profiles indicated that C20:4 (arachidonic acid) and C22:0 (docosanoic acid) resulted in an excellent potential to distinguish between IM high (*n*=13) and low gilts (*n*=12).** AUC= 0.954; and the probability graph showing the misclassification of one IM-low and IM-high.

Receiver operator curve (ROC) analysis was used to identify lipids as potential biomarkers to differentiate high and low IM or 24 h weight gain (Table 4). There were two lipids, the ether-lipid PCo(38:5) and PE(36:0)/PEp(36:6), within the M1 analysis with area under the curve (AUC) scores, indicating good (AUC=0.8-0.9) potential biomarkers to differentiate high versus low immunocrit. Within the M2 analysis, the ion transition with the tentative attribution of a saturated triacylglycerol containing 50 carbons in the three fatty acyl chains and one of them being palmitic acid [TAG(50:0)_FA 16:0] was found to have an excellent (AUC=0.9-1.0) potential as a biomarker for differentiating between low and high IM (Fig. 2). Although no single fatty acid in the FFA analysis was identified as a good or excellent biomarker in differentiating high and low IM, combining arachidonic acid (C20:4) with the very long chain saturated fatty acid, docosanoic acid (C22:0), resulted in a potential excellent biomarker, with an AUC=0.95 (Fig. 3A). The probability view (Fig. 3B) of the predictive ability of C20:4 and C22:0 markers levels for sensitivity of low IM (true-positive rate) in these samples was 92% (12 out of 13) and specificity for IM-low (true-negative rate) was 84.5% (11 out of 13).

**Table 4.**
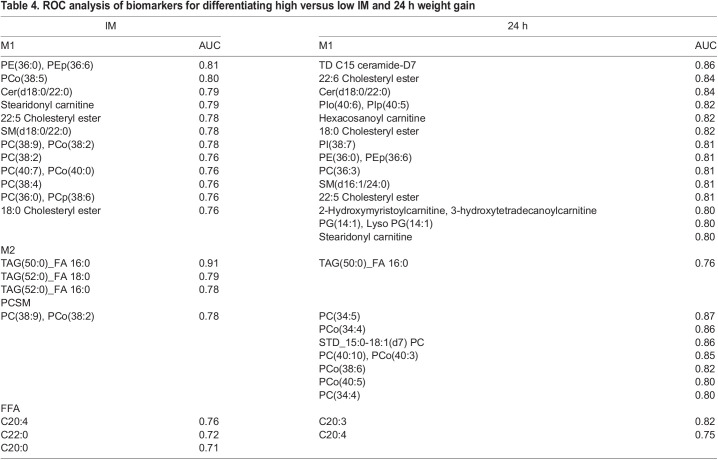
ROC analysis of biomarkers for differentiating high versus low IM and 24 h weight gain

### Relationship of gilt variables to sow colostrum composition

**Fig. 4.**
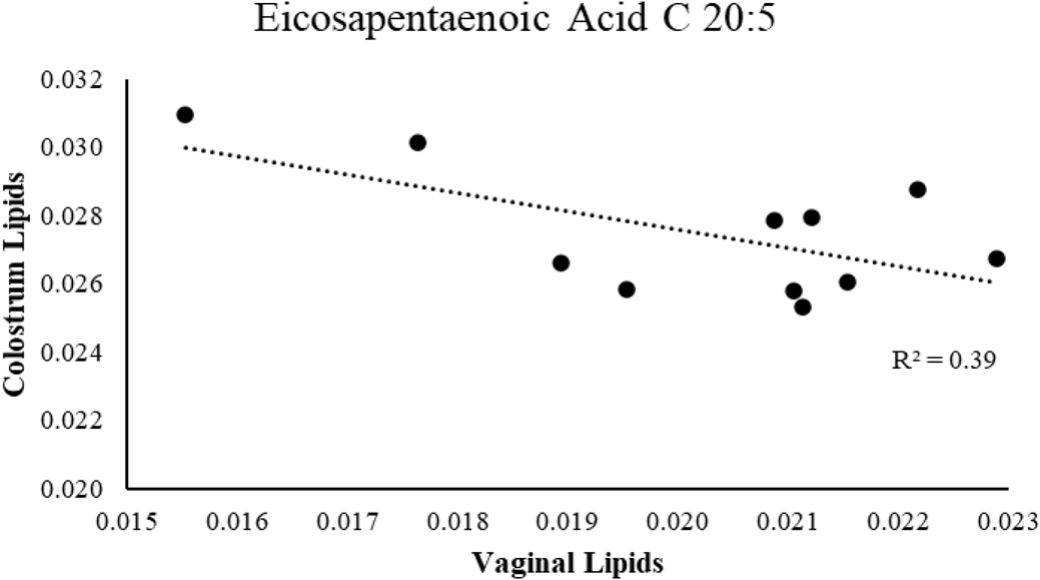
The relationship between colostrum levels of C20:5 and mean levels in vaginal swabs of littermates (*n*=51) taken at weaning.

Percent fat and protein of colostrum varied across sows with mean of fat at 14.5±6.2% and protein at 7.9±1.3%. Linear regression analysis found no significant relationship between percent fat or protein in colostrum with average birth weight, 24 h gain or IM of gilt. To investigate whether there was a relationship between colostrum lipids and abundance of lipids in vaginal swabs, correlation analysis was run between relative abundance in dam colostrum and the lipids that differentiated between high and low IM or 24 h weight gain (*P*<0.05). Both tetracosanoic acid (C24:0) and myristoleic acid (C14:1) in colostrum were positively related to relative abundance in vaginal swabs of gilts, with an R^2^ value of 0.28 and 0.22, respectively. Eicosapentaenoic acid (C20:5) in colostrum was negatively related to its level in the vaginal lipidome (R^2^=-0.39; Fig. 4). Of the 106 lipids in the M1 method (*P*<0.05), only one, SM(d16:1/24:0), was positively correlated between colostrum and vaginal lipids (R^2^=0.36).

## DISCUSSION

The primary finding of this study was the significant relationship between gilt immunocrit ratio or 24 h weight gain with the relative abundance of multiple lipids isolated from vaginal swabs taken at weaning. The distribution of lipids in vaginal cells at weaning reflects the metabolic processes that result from perinatal nutritional environment. The vagina is lined by stratified squamous epithelium. Between birth and weaning the layers of cells accumulate, and the most basal cells move toward the lumen (Harlow et al., 2019b). Thus, the morphology and developmental changes of the vagina in the first weeks postnatal reflect and preserve the history of exposures and metabolic reactions in a manner analogous to hair growth and temporal study of the bioaccumulation of substances in hair. This is significant, as it connects our previous findings of a longitudinal observational study of commercial replacement gilts that found vaginal lipidome at weaning differentiated between the most fertile animals, which went on to produce at least 26 piglets per year, from infertile gilts (Mills et al., 2021).

Lipids found in greater abundance in animals with high IM or high 24 h gain were the free fatty acids arachidonic acid (C20:4) and dihomo-γ-linolenic acid (C20:3). C20:4 was identified as a potential marker of colostrum intake in our previous work (Casey et al., 2018a), and more abundant in vaginal lipids of gilts that became highly fertile production sows versus infertile animals (Mills et al., 2021). Although ROC analysis found that C20:4 alone was only a fair biomarker to distinguish between animals with high and low IM, when it was combined with C22:0, which was more abundant in low IM, the sensitivity and specificity increased so combined the pair scored as excellent biomarkers.

PC(36:3) was more abundant in high IM or high 24 h gain groups, and it is very likely that one of the fatty acids in this lipid is an essential fatty acid, C18:2 or C18:3. In particular, the average length of the fatty acid chain in the lipid PC(36:3) is 18 carbons long and there are three unsaturated bonds, thus a combination of phosphatidyl choline with alpha-linolenic acid (C18:3) and stearic acid (C18:0) is likely. Alternatively, it could be C20:3 and C16:0, or C18:2 and C18:1, or other combinations there-in. In the M2 analysis TAG(50:0)_FA 16:0 was found more abundant in IM high versus low animals, and ROC analysis AUC score of 0.91 make it an excellent potential biomarker for distinguishing between gilts with high versus low IM.

FFA more abundant in low IM animals were the very long chain saturated fatty acids C22:0 and C20:0. In the M1 analysis the phospatidylcholine PC40:7 was found more abundant in the low versus high IM group. *De novo* discovery and screening analysis of phosphate and sphingomyelin lipids (PCSM profiles) also identified PC40:7 as a differentiating lipid between IM high and low groups and AUC score from ROC analysis indicated it as a fair biomarker (AUC=0.74). PC40:7 was previously identified as a lipid that in vaginal swabs of gilts taken at weaning was more abundant in animals found to be infertile versus highly fertile production sows (Mills et al., 2021). Similarly, the PC lipid with long-very long chain fatty acids and a high number of unsaturated binds PC40:8 was found to differentiate between high and low 24 h gain in the PCSM profiling method, being relatively more abundant in the low group. Also noteworthy was that multiple cholesteryl esters showed a negative correlation with 24 h gain and IM, among them was cholesteryl ester C18:0 which was more abundant in low IM and low 24 h gain versus high IM and gain, respectively. Cholesteryl ester C18:0 was also more abundant in gilts that went on to be infertile versus their highly fertile counterparts (Mills et al., 2021).

We have begun to recognize a pattern of the type of lipid more abundant in the low immunocrit ratio or low 24 h weight gain groups. This pattern is a greater abundance of FFA or membrane lipids with very long chain saturated fatty acids and lipids with long chain fatty acids with high levels of unsaturated bonds, similar to eicosanoids. Also, among the lipids more abundant in low IM or 24 h gain are cholesteryl esters with very long chain free fatty acids. This same pattern of lipids, higher abundance of very long chain fatty acids, highly unsaturated fatty acids and cholesteryl esters, was found to distinguish gilts that were identified as infertile from the highly fertile production sows (Mills et al., 2021). The M1 MRM profiling analysis used in the current study was generated based on the ability of selected lipids to distinguish between animals that suckled from the sow the first 48 h postnatal versus those fed milk replacer formula (Harlow et al., 2019a,b). In general, the majority of the lipids that distinguished between these groups were more abundant in the milk replacer fed group relative to the colostrum exposed group. At the time of the Harlow et al. (2019a,b) publication, we had interpreted data as the unique lipids of the replacer overwhelming the system or stimulating different metabolic pathways. However, because these same lipids continue to differentiate between animals that vary in postnatal nutrition and have never been exposed to milk replacer, we now interpret this observation as an accumulation of lipids (e.g. very long chain fatty acids) in vaginal tissue as a result of low or no colostrum intake.

Regression analysis of lipids in colostrum and mean levels in vaginal swabs across littermates found a negative relationship for C20:5 (eicosapentanoic acid) levels. C20:5 is a ligand for peroxisome proliferator activator receptor (PPAR) (Grygiel-Górniak, 2014). Milk is a rich source of lipids, and growing evidence supports PPAR mediated metabolic pathways must be initiated shortly after birth so neonates can catabolize fats (Hashimoto and Ogawa, 2018; Zhao et al., 2023; Shim et al., 2018; Yu et al., 1998). Expression of PPARα and PPARAγ was found significantly higher in peripheral blood mononuclear cells of breastfed versus formula-fed infants (Cheshmeh et al., 2020, 2022). In rodents, postnatal intake of milk demethylates DNA regions that encode for fatty acid beta-oxidation regulatory genes in a PPARα dependent manner (Ehara et al., 2015). Supplementing milk replacer fed to neonatal pigs with synthetic PPAR agonist increased peroxisome β-oxidation in hepatic tissue (Yu et al., 2001). PPAR mediated pathways are important to regulation of pluripotent stem cell populations (Nakashima et al., 2018), and thus PPARs may function in nutritional programming.

Within mammalian cells, fatty acid metabolism involves the coordination between peroxisomal and mitochondrial β-oxidation. Peroxisomal β-oxidation reduces very long chain and ultra-long chain fatty acids (i.e. >C20) and cholesteryl esters and eicosanoids are metabolized in peroxisomes. It is these lipids that seem to be potentially accumulating in the vaginal tissue of gilts with low or no colostrum intake. Individuals with peroxisomal disorders have buildup of very long-chain fatty acids, eicosanoids and cholesteryl esters in their tissues (Grønn et al., 1990; Lodhi and Semenkovich, 2014; Nenicu et al., 2009). Peroxisomes play central roles in lipid homeostasis (Farr et al., 2016) and removal of lipid metabolites that mediate inflammation. There is an increase in number of peroxisomes over the first day postnatal (Laging et al., 1990). The accumulation of eicosanoids or long chain fatty acids may also elicit a very different cellular signaling environment within a tissue as well as inflammation. Inflammation alone can affect stem cell proliferation and progenitor cell differentiation pathways (Kizil et al., 2015). Thus, this may also explain the connection between level of colostrum intake and body composition, as well as factors like long term fertility and milk production capacity of animals, as the number of epithelial cells in the gland determines milk yield. Together these data support the need for further research to determine whether colostrum lipids such as C20:5 initiate PPAR mediated signaling, peroxisome biogenesis and beta-oxidation metabolism of lipids in neonatal tissues.

In addition to the potential mechanistic insight of these data to neonate development and metabolism, the findings support that vaginal lipid profiles can potentially identify animals with low colostrum intake. The strong link between colostrum intake and long-term fertility, make colostrum intake level an ideal first selection tool. However, there is no direct measure of colostrum intake, and so currently immunocrit ratio and equations based on 24 h weight gain are used to estimate intake. A study of the value of IM in a commercial setting found the ratio is best able to identify animals that did not nurse, as indicated by very low ratios (Vallet et al., 2013). More work needs to be done in this area, but the good predictive value of fatty acid profiles to differentiate high and low IM demonstrate a potential of at least replacing IM analysis, which requires a blood sample at 24 h of age and is not practical in a commercial setting.

In conclusion, weight gain the first 24 h postnatal and immunocrit ratio reflect perinatal nutritional environment which affects long-term growth, development and fertility of swine. Lipid profiles were significantly different between gilts with high versus low immunocrit ratio and 24 h weight gain and the abundance of multiple lipids in vaginal swabs taken at weaning showed a linear relationship with immunocrit ratio and 24 h gain. Lipid profiles of low colostrum intake animals had greater abundance of very long chain fatty acids, lipids with high levels of unsaturation, and cholesteryl esters, which are metabolized in peroxisomes indicating potential dysfunction of peroxisomes and β-oxidation, and thus warrants further research in this area.

## MATERIAL AND METHODS

### Animals

Prior to conducting studies, all animal use protocols were reviewed and approved by Purdue University's Institutional Animal Care and Use Committee (Protocol # 1907001920). Gilts (*n*=60) selected for the study were from eleven litters (*n*=3-7 gilts/litter) of Duroc and Landrace sired sows that farrowed between May 6-7, 2021 on Purdue University Animal Sciences Research and Education Center Swine Farm. Gilts were handled as per standard farm protocols until weaning: piglets were weighed and processed (tails docked, ear notched, and vaccines given) at ∼24 h postnatal (PN) and cross-fostered after 24 h PN. Litters were standardized to 13 piglets per litter. Data collected included farrowing time, birth litter size, birth weight, nursing litter size and any cross fostering to standardized litters, weaning weight and date. Gilt weight was recorded at birth, 24 h PN, and every week until 3 weeks PN. Blood samples were taken from the jugular vein of the gilts (*n*=60) at 24 h postnatal with blood plasma isolated and frozen at −20° C for immunocrit (IM) analysis. Fifty-three gilts survived until weaning (21±1 d) and were moved to the nursery at weaning.

Prior to moving the piglets into the nursery on weaning day, vaginal swabs were taken for lipidome analysis using a human pap smear cytology brush (Cytobrush Plus GT Scored Gentle touch tip cell collector, Trumbull, CT, USA). To collect the vaginal sample, the external genitalia of the gilts was sprayed with 70% ethanol and wiped clean with a paper towel. Cytology brushes were then inserted as far as possible into the vaginal canal and turned clockwise five times. Vaginal swabs were taken in duplicate and placed in 15 mL polypropylene conical tubes (Corning™ Falcon™, Corning, NY, USA). The samples were stored at −20° C until the completion of the live animal portion of the study. Samples were transported on ice to Purdue University and stored at −80° C until lipidome analysis.

### Immunocrit analysis

At 24 h PN, blood was collected from the jugular vein using a vacutainer tube. Blood samples were centrifuged for 15 min at 10,000 rpm. Plasma was removed and aliquots were placed in 1.8 µl microfuge tubes. Plasma was frozen on site at −20° C and transported to the lab on ice. A 40% ammonium sulfate solution was made with dd H_2_0. Plasma was thawed on ice, and equal volumes of plasma and 40% ammonium sulfate were mixed together (1:1). Capillary tubes (*n*=3 technical replicates) were filled with plasma-ammonium sulfate and sealed. Capillary tubes were centrifuged for 10 min at 12,000 g in a MX12 Micro Combo Centrifuge (LW Scientific, Lawrenceville, GA, USA). Immunocrit ratio was determined using a digital caliper, and by measuring total length of solution-precipitate and length of precipitate alone. Finally, the length of precipitate was divided by the total length of solution-precipitate. Data are reported as the average of three technical replicates.

### Categorization of gilts into 24 h gain and IM level groups

Gilt 24 h gain (*n*=53) or IM levels (*n*=51) were used to categorize gilts into one of four groups: high, medium-high, medium-low, and low based on weight gain or ratio (Fig. 1). Assigned groups were determined by the median of each variable (24 h gain or IM), with half of the group placed in the high and the other half in the low. Within the high and low group, another median was determined to split the group into high and medium-high or low and medium-low.

#### Colostrum percent fat and protein analysis

The creamatocrit method was used to determine percent fat of sow colostrum (Lucas et al., 1978). Milk protein was measured using a Bradford assay (Fisher Scientific, Waltham, MA, USA) following diluting milk samples 1:100 in phosphate buffered saline (PBS). Each sample was analyzed in triplicate.

### Lipid extraction and rapid lipid screening using the multiple reaction monitoring (MRM) profiling method

Lipid extraction of material collected from swabs and the lipid rapid screening were carried out as described in Mills et al. (2021). Briefly, lipids and cellular material were removed from swabs by vortexing cytology brush head in 500 µl of ddH2O, and extracted using the Bligh–Dyer method (Bligh and Dyer, 1959). Following extraction, lipids were dried for 8 h in a speed vacuum. Pellets were resuspended to a stock solution with 200 µl of acetonitrile (ACN), methanol (MeOH) and ammonium acetate 300 mM (NH_4_Ac) with a ratio of (3:6.65:0.35, respectively). The stock solution was vortexed and 5 µl of it was placed in new tubes and diluted 10×with 45 µl of the ACN: MeOH: NH_4_Ac solvent mixture.

The method cannot discriminate isomeric lipids. MRM profiling was conducted by injecting 8 µL of the diluted lipid extract stock solution using a micro autosampler (G1377A) in a QQQ6410 triple quadrupole mass spectrometer (Agilent Technologies, San Jose, CA, USA) equipped with an ESI ion source. Methods called M1, M2, phosphatidylcholine and sphingomyelin lipids (PCSM), and free fatty acids (FFA) were used in the lipidome profiling. Description of the methods can be found in Mills et al. (2021).

### Data analysis of the lipid profiling using MetaboAnalyst 5.0 and lipid structural identification

The relative abundance of each MRM corresponding to the composition of the sample for the lipids monitored were calculated as described previously (Mills et al., 2021). Since the biological material recovered by swabbing is not the same for each sample, no quantitative analysis was pursued. Briefly, the relative ion intensity of an MRM within each sample was calculated after removing MRM ion pairs with an absolute intensity of 1.3-fold less than the level of the blanks analyzed. Relative ion intensity was determined by dividing the intensity by the sum of intensities of all lipids within a sample, and within a screening method. Lipid Maps (http://www.lipidmaps.org/) was used to attribute tentative lipid classes based on functional group and biological information (Reis et al., 2023). Relative intensity ion pairs were uploaded to Metaboanalyst 5.0 and the data was normalized using the auto scaling feature. Correlation analysis was used to determine if lipids abundance varied in relation to IM or 24 h weight gain. Student *t*-tests were used to identify lipid profiles that distinguished between groups. Raw *P*-value <0.05 was used to identify lipids different between high and low groups as specified. Univariate receiver operating characteristic (ROC) curve analysis with calculated area-under-the-curve (AUC) value was used to determine potential biomarkers that discriminated based on high versus low IM or 24 h gain, with AUC scale reflecting biomarker potential as: excellent=0.9–1.0; good=0.8–0.9; fair=0.7–0.8; poor=0.6–0.7; fail=0.5–0.6 (Xia et al., 2013).

## Supplementary Material

10.1242/biolopen.060044_sup1 Supplementary information
