# Supplementary material for: Low colostrum intake results in potential accumulation of peroxisome lipid substrates in vaginal tissue of 3-week-old gilts
Source: Biol Open. 2023 Aug 11;12(8):bio060044. doi: 10.1242/bio.060044 (PMC10434361; doi:10.1242/bio.060044)
Supplement: Supplementary information [file biolopen-12-060044-s1.pdf]

Table S1.

Click here to download Table S1

Table S2.

Click here to download Table S2

Table S3.

Click here to download Table S3

Table S4.

Click here to download Table S4

Biology Open • Supplementary information

Table S5.

Click here to download Table S5

Table S6.

Click here to download Table S6

Table S7.

Click here to download Table S7

Table S8.

Click here to download Table S8

Biology Open • Supplementary information
